# Quality assurance incorporating artificial intelligence-generated reference contours in a phase II radiotherapy trial

**DOI:** 10.1016/j.phro.2026.100949

**Published:** 2026-03-18

**Authors:** Wonhyeong Lee, Yeon-Joo Kim, Jin Hee Kim, Sung-Ja Ahn, Jong Hoon Lee, Younghee Park, Jin Hwa Choi, Jin-ho Song, Yoonsun Chung

**Affiliations:** a Department of Radiation Oncology, National Cancer Center, Goyang, the Republic of Korea; b Department of Nuclear Engineering, Hanyang University, Seoul, the Republic of Korea; c Department of Radiation Oncology, Keimyung University Dongsan Hospital, Daegu, the Republic of Korea; d Department of Radiation Oncology, Chonnam National University Medical School, Gwangju, the Republic of Korea; e Department of Radiation Oncology, St. Vincent’s Hospital, The Catholic University of Korea College of Medicine, Seoul, the Republic of Korea; f Department of Radiation Oncology, Ewha Woman’s University College of Medicine, Seoul, the Republic of Korea; g Department of Radiation Oncology, Chung-Ang University College of Medicine, Seoul, the Republic of Korea; h Department of Radiation Oncology, Seoul St. Mary’s Hospital, College of Medicine, The Catholic University of Korea, Seoul, the Republic of Korea

**Keywords:** Breast cancer, Regional nodal irradiation, Multi-institutional study, Dummy run, Neoadjuvant chemotherapy

## Abstract

**Background and purpose:**

A dummy run quality assurance was conducted for a prospective phase II trial of tailored radiotherapy (RT) according to the response after neoadjuvant chemotherapy (NAC) followed by surgery in locally advanced breast cancer (RTaNAC). Since inter-institutional variations in dose distributions can impact clinical outcomes, this dummy run aimed to develop an RT plan protocol to minimize these variations.

**Material and methods:**

This study involved computed tomography images from three clinical scenarios: RT with no lymph node (LN) boost (Scenario 1), LN boost up to 60 Gy_3.5_, equivalent dose in 2 Gy fractions (EQD2) with α/β = 3.5 Gy (Scenario 2), and LN boost up to 66 Gy_3.5_ (Scenario 3). Seven institutions developed RT plans under a two-step process: first according to each institution’s policies (Step 1) and then using additional reference information, including artificial intelligence (AI) auto-contoured structures and LN boost target volume information (Step 2). Dose-volume histograms for breast and regional nodal areas were analyzed between the two steps.

**Results:**

Inter-institutional variation observed in Step 1 improved in Step 2 for breast and regional nodal areas. Specifically for Scenario 1, dose coverage for the nodal clinical target volumes of the axillary level I LN improved from 57.5% to 97.5% (p-value = 0.075), and that of the supraclavicular LN improved from 75.6% to 88.8% (p-value = 0.046).

**Conclusion:**

Variations in dose/volume metrics among institutions were mitigated by AI auto-contoured structures and LN boost target volume information. Through this dummy run, the participating institutions reached a consensus on an RT plan protocol to support multi-institutional expansion of RTaNAC.

## Highlights

• Tailored radiotherapy for locally advanced breast cancer was investigated.
• A dummy run procedure minimized variations across seven institutions.
• Dose coverage for regional nodal areas increased by up to 40%
• Auto-contoured structures and lymph node boost target volume mitigated variations.
• A multi-institutional consensus on radiotherapy plan protocol was established.

## Graphical abstract

**Figure:**
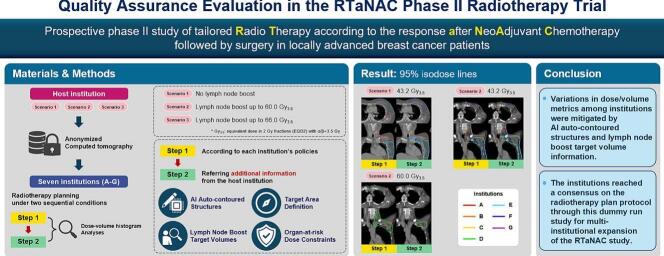

## 1 Introduction

The National Comprehensive Cancer Network (NCCN) clinical practice guidelines in oncology recommend a supplemental radiotherapy (RT) boost may be delivered to grossly involved or enlarged lymph nodes (LNs) that have not been surgically removed in breast cancer patients [1]. In our previous retrospective study, we observed that patients who received dose-escalation of hypofractionated regional nodal irradiation (RNI) faced a higher risk of developing lymphedema and shoulder stiffness compared with those who received standard dose hypofractionated RNI [2]. However, results from the National Surgical Adjuvant Breast and Bowel Project (NSABP) B-18 and B-27 trials demonstrated that the 10-year cumulative regional recurrence rate was low (0%–2.4%) in patients with cT1-3N1 (clinical stage) and ypN0 (pathological stage after neoadjuvant therapy) disease after neoadjuvant chemotherapy (NAC) and breast-conserving surgery, even though RNI was omitted [3]. This indicates that breast cancer patients with LN metastases at diagnosis who achieve complete remission after NAC are expected to have low recurrence rates regardless of supplemental LN boost. We initiated a prospective Phase 2 study of tailored RT according to the response after NAC followed by surgery in patients with axillary level II-III nodes and/or internal mammary node (IMN) and/or supraclavicular lymph node (SCN) metastasis at diagnosis (RTaNAC, Supplementary Fig. S1). We hypothesized that among patients who achieved complete remission after NAC, those who received a standard dose would have no difference in recurrence rates and reduced lymphedema incidence compared to those who received a supplemental LN boost. We also planned to evaluate lymphedema rates in patients who did not achieve complete remission after NAC, to whom we delivered a tailored LN boost according to NAC response.

Although the International Commission on Radiation Units and Measurements (ICRU) published the ICRU-83 Report to provide a standardized guideline for intensity-modulated radiation therapy (IMRT) [4], [5], there remains inconsistency in delivered IMRT doses among institutions [6]. In this context, several multi-institutional studies involving IMRT have conducted dummy run quality assurance (QA) programs to ensure the reliability of collected clinical data by evaluating and mitigating inter-institutional variations [7], [8], [9].

Since RTaNAC is a multi-institutional IMRT study, inter-institutional variations in dose distributions among RT plans could significantly affect clinical outcomes. Therefore, we conducted a dummy run before expanding RTaNAC into a multi-institutional study. Through a two-step dummy run procedure, this study aimed to identify variations in dose/volume metrics among institutions and evaluate what additional information could improve these variations. These results were used to develop an RT plan protocol for RTaNAC, ensuring consistency in dose distributions.

## 2 Materials & methods

This study was conducted as a dummy run quality assurance for the RTaNAC clinical trial, which was registered with the Clinical Research Information Service (CRIS) (Registration No. KCT0009061). The specific protocol for this dummy run was approved by the Institutional Review Board (IRB) of the National Cancer Center, Korea (Approval No. NCC2023-0225).

### 2.1 Questionnaire

Seven participating institutions completed a questionnaire before the dummy run. The following treatment plan information was gathered: treatment unit, treatment planning system, photon beam energy, treatment technique, dose calculation algorithm, planning target volume (PTV) margin, and use of artificial intelligence (AI) auto-contouring according to institutional policy.

### 2.2 Clinical scenarios

This study involved RT plans for three clinical scenarios (Scenarios 1–3) based on computed tomography (CT) images of breast cancer patients. The host institution provided anonymized CT images in Digital Imaging and Communications in Medicine (DICOM) files to the participating institutions. All scenarios represented patients with axillary level II-III nodes and/or IMN and/or SCN metastasis at diagnosis who underwent neoadjuvant chemotherapy followed by surgery. The RT plans were composed of a base RT plan, which prescribed a standard dose to the whole breast and regional nodal area, and an LN boost RT plan, which prescribed a boost dose to regional nodal areas. The standard dose in all scenarios was 43.2 Gy/16 fractions, with varying LN boost doses. All scenarios involved left breast cancer patients, taking into consideration that the heart is a critical organ at risk (OAR) in breast cancer treatment planning.

Scenario 1: Left breast invasive ductal carcinoma (IDC), cT3N2 (axillary level I-II nodes), with complete remission on both imaging and pathology, and no LN boost.

Scenario 2: Left breast IDC, cT1N3 (axillary level I, II, III node), without residual LNs observed on CT scan for RT. For this scenario, a sequential LN boost plan of 12 Gy/6 fractions was prescribed to the initial LN beds, defined as the area where metastatic LN existed at the time of diagnosis but were not surgically removed, resulting in up to 60 Gy_3.5_, equivalent dose in 2 Gy fractions (EQD2) with α/β = 3.5 Gy.

Scenario 3: Left breast IDC, cT3N3 (axillary level I, II, III node, and SCN), with residual LNs observed on CT scan for RT. For this scenario, a sequential LN boost of 12 Gy/6 fractions to the initial LN beds and 15.6 Gy/6 fractions to the residual LNs was prescribed, resulting in up to 66 Gy_3.5_ to the residual LNs.

### 2.3 Two-step dummy run procedure

**Fig. 1:**
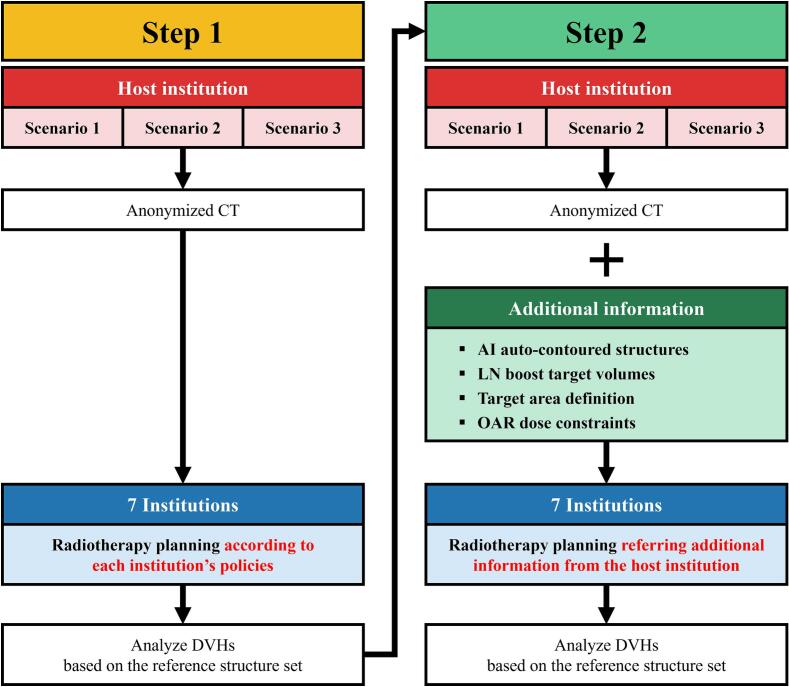
Flow chart of the dummy run. Step 1, RT planning according to each institution’s policies; Step 2, RT planning incorporating additional information from the host institution. Abbreviations: CT, computed tomography; DVH, dose-volume histogram; AI, artificial intelligence; LN, lymph node; OAR, organ at risk.

As shown in Fig. 1, the seven participating institutions developed RT plans from the three scenarios under a two-step sequential process. Step 1 involved RT planning according to each institution’s policies. For each scenario, the host institution provided anonymized CT images, dose/fraction prescriptions, and minimal boost LN structures. The boost LN structures, including the initial LN beds (clinical target volume, CTV) and residual LNs (gross tumor volume, GTV), were contoured by a radiation oncologist at the host institution. In Step 2, however, the host institution requested that participating institutions incorporate additional information into their RT plan.

The host institution provided AI auto-contoured CTV and OAR structures using a customized version of the commercial AVIEW AI auto-contouring engine (Coreline Soft Inc., Seoul, Korea) [10], [11], [12]. Through transfer learning at the host institution, the AI model was optimized to contour additional structures and accommodate diverse patient postures for the RTaNAC study. Supplementary Fig. S2 illustrates the AI auto-contoured CTVs provided. The CTVs included the left breast, axillary levels I-III nodes, interpectoral node, and SCN based on both the European Society for Radiotherapy and Oncology (ESTRO) and the Radiation Therapy Oncology Group (RTOG) consensus guidelines [13], [14]. The OARs included the heart, ipsilateral lung, contralateral breast, and ipsilateral shoulder joint. The provided CTVs were intended as reference structures, and participating institutions were allowed to use their own contours.

The host institution provided boost LN PTV structures to all participating institutions. As shown in Supplementary Fig. S2, the boost LN PTVs included a 5–7 mm margin around the corresponding CTV or GTV.

The host institution defined the regional nodal areas to be irradiated in each scenario. A standard dose of 43.2 Gy_3.5_ was specified for the left breast, axillary levels I-III nodes, interpectoral node, and SCN. The boost doses of 60.0 and 66.0 Gy_3.5_ were assigned to the PTVs of the initial LN beds and residual LNs, respectively.

From Step 1, the host institution collected RT plans from all participating institutions and extracted OAR doses based on AI auto-contoured structures. Using this data, the host institution provided OAR dose constraints for each scenario in Step 2. Regarding the brachial plexus, since the AI auto-contouring engine used in this study could not generate this structure, the dose constraint was indirectly addressed by restricting the maximal dose to the whole body to less than 70 Gy_3.0_.

### 2.4 Data analysis

We analyzed dose-volume histograms (DVHs) for targets and OARs to evaluate dose distributions across the participating institutions. For the targets, we calculated the minimum dose delivered to 95% of the structure (D95%) relative to the prescribed dose for the left breast (Breast Lt) and the axillary levels I, II, III node (CTVn-L1-L3), interpectoral lymph node (CTVn-intpect), and SCN (CTVn-SCN (ESTRO and RTOG)) regional nodal CTVs. The D95% values were also calculated for the boost PTVs (PTVb-12 Gy, 15.6 Gy) in scenarios with LN boost. For the OARs, we determined the mean dose to the heart, the volume receiving 20 Gy (V20 Gy) for the left lung, the volume receiving 5 Gy (V5 Gy) for the right breast, and the volume receiving 15 Gy (V15 Gy) for the left shoulder joint. All DVHs were extracted from the AI auto-contoured structures.

Calculated DVHs were expressed as median (range). Differences in median dose between Steps 1 and 2 were evaluated using the Wilcoxon signed-rank test. Boxplots of dose/volume metrics were created for each scenario and structure. The boxplots display minimum, maximum, first quartile (Q1), third quartile (Q3), and median values across all participating institutions. Values more than 1.5 times the interquartile range (IQR, defined as Q3–Q1) from Q1 or Q3 were defined as outliers.

We calculated Fleiss’s kappa value and Dice Similarity Coefficient (DSC) for 95% isodose lines in each scenario and prescribed dose from participating institutions, and compared them between Steps 1 and 2. The Fleiss’s kappa value and the DSC were calculated using the Computational Environment for Radiotherapy Research (CERR) in MATLAB as metrics to assess the agreement and similarity of the isodose lines between institutions [15], [16], [17]. For the DSC, we compared the isodose lines from each institution to those of the host institution, presenting the results as medians. Differences in median DSCs between Steps 1 and 2 were evaluated using the Wilcoxon signed-rank test.

All statistical analyses were performed using Python (version 3.12). Boxplots were created using the Matplotlib (version 3.8.2) library, and the Scipy (version 1.13.1) library was used for the Wilcoxon signed-rank tests. Any p-value under 0.05 was considered statistically significant.

## 3 Results

Table 1 summarizes the treatment plan information of the seven participating institutions from the questionnaires before the dummy run. Four institutions added PTV margins to CTVs, while the remaining three added no PTV margin. None of the institutions used any AI auto-contouring engine for target structures in their current treatment planning policies. However, for OAR structures, all but one institution used commercial AI auto-contouring software in treatment planning.

**Table 1:** Treatment plan information.

Institution	TreatmentUnit	Treatment Planning System	Photon Beam Energy	Treatment Technique	Dose Calculation Algorithm	PTV margin*	Use of AI auto-contouring engine according to institutional policy	
							Target	OAR
A	Accuray, Radixact	Precision 3.3.1.3	6 MV	TOMO	Convolution-Superposition	Uniform (5 mm)	No	AccuContour (Manteia) Limbus (Limbus AI Inc.)
B	Varian, VitalBeam	Eclipse v15.5	6 MV	IMRT (7 Fields)	AAA	Uniform (5–6 mm)	No	AccuContour (Manteia)

C	Varian, Truebeam	Eclipse v15.6	6 MV	VMAT (4 Arcs)	AAA	Variable(Post:3 mm, others: 5 mm)	No	AccuContour (Manteia)
D	Varian, Halcyon	Eclipse v16.01	6 MV	VMAT (5 Arcs)	AAA	No margin	No	Oncostudio (OncoSoft Inc.)
E	Accuray, Radixact	Precision 1.1.1.1	6 MV	TOMO	Convolution-Superposition	No margin	No	AccuContour (Manteia)
	Elekta, VersaHD	Monaco 5.51.10	6 MV, 10 MV	IMRT (3–4 Fields) VMAT (1 Arc)	Monte Carlo			
F	Varian, 21ix	Eclipse 13.7	6 MV	IMRT (6 Fields)	AAA	No margin	No	No

G	Varian, Halcyon	Eclipse 16.1	6 MV	VMAT (2–4 Arcs)	AAA	Uniform (3 mm)	No	AccuContour (Manteia)

*PTV for LN boost target was provided by the host institution with a 5 mm margin.

Abbreviations: PTV, planning target volume; LN, lymph node; OAR, organ at risk; MV, megavoltage; TOMO, helical tomotherapy; IMRT, intensity-modulated radiation therapy; VMAT, volumetric-modulated arc therapy; AAA, anisotropic analytic algorithm.

**Fig. 2:**
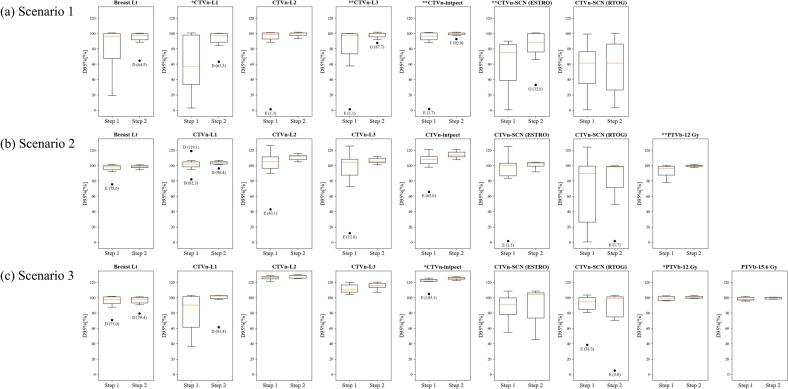
Boxplots of dose-volume histogram parameter distributions in target volumes across the participating institutions for Step 1 and Step 2. (a) Scenario 1, (b) Scenario 2, and (c) Scenario 3. The boxplots contain minimum/maximum, first (Q1)/third (Q3) quartile, and median values. Values that are more than 1.5 interquartile range (IQR, Q3−Q1) away from Q1 or Q3 are defined as outliers, represented by a black dot (•) along with an institution identifier and corresponding value. Single (*) and double (**) asterisks denote marginal (p-value <0.1) and statistical significance (p-value < 0.05), respectively. Abbreviations: Lt, left; CTVn, nodal clinical target volume; L1-3, axillary level 1–3 node; Intpect, interpectoral lymph node; SCN, supraclavicular node; ESTRO, European Society for Radiotherapy and Oncology; RTOG, Radiation Therapy Oncology Group; D95%[%], minimum dose delivered to 95% of the structure, relative to the prescribed dose; PTVb, boost planning target volume.

The boxplots in Fig. 2 illustrate the DVH parameter distributions across the participating institutions for Steps 1 and 2. For most target DVH parameters, the IQR decreased in Step 2 compared to Step 1. Of the outliers observed in the target boxplots in Step 1, 77% moved into the IQR in Step 2. Although new outliers were observed in Step 2, these were found to be driven by the narrowed IQRs. Supplementary Fig. S3 shows that participating institutions achieved most of the OAR dose constraints in Step 2.

As detailed in Table 2, Step 2 demonstrates improvements in the DVH parameters. In Scenario 1, median D95% for CTVn-L1 improved by 40% (from 57.5% in Step 1 to 97.5% in Step 2, p-value = 0.075). Median D95% for CTVn-SCN (ESTRO) showed a statistically significant improvement by 10% (from 75.6% to 88.8%, p-value = 0.046). Similarly, median D95% for both CTVn-L3 and CTVn-intpect in Scenario 1 significantly increased from 97.8% and 96.6% to 100.1% and 100.3%, respectively (p-value = 0.046). A similar trend was observed in Scenario 2, where median D95% for PTVb-12 Gy also showed a significant increase from 96.2% to 99.9% (p-value = 0.031). In Scenario 3, median D95% for both CTVn-intpect and PTVb-12 Gy showed marginally significant improvements (p-value = 0.075).

**Table 2:** Comparison of dose-volume histogram parameters between Step 1 and Step 2.

			Scenario 1			Scenario 2			Scenario 3		
			Step 1	Step 2	p-value	Step 1	Step 2	p-value	Step 1	Step 2	p-value
Target	Breast Lt	D95% [%]	96.8(19.0–100.7)	97.5(64.5–100.3)	*0.463*	98.5(75.6–101.5)	98.4(94.5–101.3)	*0.463*	97.3(71.0–101.8)	97.9(79.4–101.1)	*0.463*
	CTVn-L1	D95% [%]	57.5(3.0–100.7)	97.5(63.3–100.6)	*0.075*	102.2(82.3–119.1)	104.2(96.4–106.8)	*0.345*	90.5(36.7–103.4)	102.2(61.4–103.4)	*0.116*
	CTVn-L2	D95% [%]	99.1(1.3–101.3)	100.3(93.4–101.8)	*0.173*	104.9(43.1–125.9)	111.8(104.7–115.9)	*0.249*	126.2(121.6–128.6)	128.6(124.9–130.1)	*0.116*
	CTVn-L3	D95% [%]	97.8(1.1–100.1)	100.1(87.7–101.7)	*0.046*	104.7(12.0–125.6)	105.7(100.9–112.3)	*0.249*	111.3(104.3–120.0)	115.7(106.9–120.6)	*0.173*
	CTVn-intpect	D95% [%]	96.6(1.7–101.6)	100.3(92.9–101.8)	*0.046*	108.1(65.8–121.1)	113.6(107.6–121.1)	*0.116*	123.1(105.1–125.6)	125.6(122.7–127.2)	*0.075*
	CTVn-SCN (ESTRO)	D95% [%]	75.6(0.6–90.1)	88.8(32.8–100.8)	*0.046*	100.6(1.5–125.1)	102.4(92.0–104.7)	*0.249*	90.9(55.1–108.8)	104.4(45.7–108.8)	*0.753*
	CTVn-SCN (RTOG)	D95% [%]	61.6(0.5–99.6)	61.6(3.2–100.5)	*0.753*	89.9(0.7–124.2)	98.3(1.7–99.7)	*0.600*	94.5(38.3–103.6)	99.4(5.0–102.9)	*0.345*
	PTVb-12 Gy	D95% [%]	−	−	*−*	96.2(78.1–100.4)	99.9(97.3–101.7)	*0.031*	99.5(95.9–102.6)	100.8(99.1–103.2)	*0.075*
	PTVb-15.6 Gy	D95% [%]	−	−	*−*	−	−	*−*	98.2(95.8–102.0)	100.3(98.0–100.9)	*0.345*

OAR	Heart	Mean [Gy]	6.1(1.7–10.0)	5.9(4.5–8.6)	*0.753*	4.3(1.0–8.5)	4.8(2.0–7.6)	*0.917*	4.9(3.0–11.3)	5.3(2.6–12.4)	*0.753*
	Lung Lt	V20 Gy [%]	14.3(4.4–27.5)	23.9(11.8–26.3)	*0.075*	15.6(12.3–25.7)	18.7(13.4–26.5)	*0.043*	21.9(12.6–35.6)	27.7(10.5–41.6)	*0.917*
	Breast Rt	V5 Gy [%]	8.4(0.0–27.4)	13.9(2.6–20.0)	*0.917*	6.1(0.0–30.1)	6.8(0.0–17.6)	*0.500*	7.6(0.6–17.1)	11.8(1.5–13.9)	*0.753*
	Shoulder joint Lt	V15 Gy [%]	59.4(0.0–89.8)	46.2(15.7–89.8)	*0.917*	43.1(6.0–83.1)	51.8(22.8–83.1)	*0.463*	84.0(46.6–94.3)	67.8(45.0–94.3)	*0.249*

The values are shown as median(range).

*Abbreviations*: D95%[%], minimum dose delivered to 95% of the structure relative to the prescribed dose; CTVn, nodal clinical target volume; L1-3, axillary level 1–3 node; Intpect, interpectoral lymph node; SCN, supraclavicular node; ESTRO, European Society for Radiotherapy and Oncology; RTOG, Radiation Therapy Oncology Group; PTVb, boost planning target volume; OAR, organ at risk; Lt, left; Rt, right; V20 Gy, volume receiving 20 Gy; V5 Gy, volume receiving 5 Gy; V15 Gy, volume receiving 15 Gy.

**Fig. 3:**
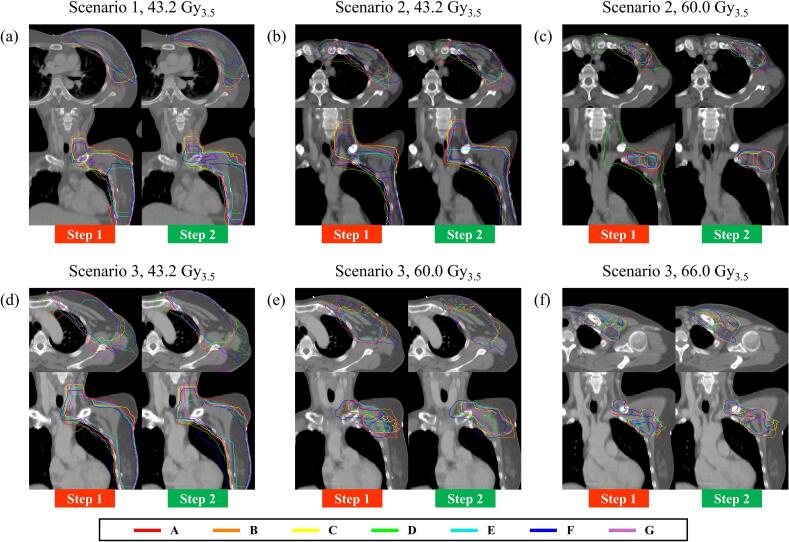
95% isodose lines for each participating institution by scenario and prescribed dose. Each set of CT images includes an axial view (top) and a coronal view (bottom), with comparisons between Step 1 (left) and Step 2 (right). Abbreviation: Gy_3.5_, equivalent dose in 2 Gy fractions (EQD2) with α/β = 3.5 Gy.

Fig. 3 illustrates 95% isodose lines from the participating institutions for each scenario and prescribed dose. The 95% isodose lines for each participating institution are delineated by color. Table 3 presents comparisons of quantitative metrics for the 95% isodose lines between Steps 1 and 2. For almost all 95% isodose lines, Fleiss’s kappa values and median DSCs increased in Step 2 compared to Step 1. For the 60 Gy_3.5_ 95% isodose lines in Scenario 2, both Fleiss’s kappa and median DSC improved from 0.43 to 0.73 and from 0.63 to 0.76 (p-value = 0.031), respectively.

**Table 3:** Isodose line agreement and similarity.

95% Isodose line			Volume [cm^3^]	Fleiss’s kappa	Dice Similarity Coefficient	
			Mean (STD)		Median (range)	p-value
Scenario 1	43.2 Gy_3.5_	Step 1	1255(339)	0.70	0.73(0.56–0.82)	*0.219*
		Step 2	1281(306)	0.74	0.75(0.68–0.85)	
Scenario 2	43.2 Gy_3.5_	Step 1	618(141)	0.71	0.78(0.65–0.81)	*0.156*
		Step 2	637(141)	0.75	0.79(0.64–0.86)	
	60.0 Gy_3.5_	Step 1	75(74)	0.43	0.63(0.37–0.67)	*0.031*
		Step 2	66(13)	0.73	0.76(0.72–0.87)	
Scenario 3	43.2 Gy_3.5_	Step 1	1331(205)	0.77	0.82(0.74–0.84)	*0.156*
		Step 2	1361(200)	0.78	0.81(0.76–0.87)	
	60.0 Gy_3.5_	Step 1	253(42)	0.73	0.79(0.70–0.83)	*0.219*
		Step 2	292(50)	0.75	0.80(0.70–0.88)	
	66.0 Gy_3.5_	Step 1	122(26)	0.67	0.72(0.53–0.87)	*0.438*
		Step 2	152(37)	0.69	0.78(0.59–0.86)	

*Abbreviations*: STD, standard deviation; Gy_3.5_, equivalent dose in 2 Gy fractions (EQD2) with α/β = 3.5 Gy.

## 4 Discussion

This study demonstrated that inter-institutional variation in dose distribution can be reduced by integrating AI auto-contoured structures and LN boost target volume information into RT planning. This benefit was particularly pronounced in Scenario 1, where metastasis was confined to axillary level I-II nodes at diagnosis. In this limited initial nodal burden scenario, some institutions fell short of the standard dose to the regional nodal areas in Step 1, especially for axillary levels I, III, and SCN. These discrepancies were mitigated in Step 2, leading to improved inter-institutional consistency in dose distributions and regional nodal coverage. In contrast, Scenarios 2 and 3 presented advanced nodal metastases, involving axillary level III and SCN in addition to axillary levels I-II. For these scenarios with extensive initial nodal burden, most participating institutions successfully achieved standard dose coverage even in Step 1. Across all regional nodal areas in Scenarios 2 and 3, with the exception of CTVn-SCN (RTOG) for Scenario 2, median D95% had already exceeded 90% of the standard dose in Step 1 (Table 2).

This study validated that AI auto-contouring significantly reduced inter-institutional variation in planning and dosimetry, especially for regional nodal area targets. For CTVn-L1 and CTVn-SCN (ESTRO), dose coverage in Scenario 1 was insufficient compared to Scenarios 2 and 3. In Step 2, tailored contours generated by the AI auto-contouring engine were provided to all participating institutions, resulting in improved dose coverage. This suggests that patient-specific variations in dose distributions may be overcome through AI auto-contouring. The additional information provided by the host institution, such as the target area definition and LN boost target volumes, also reduced variation in dose distribution. The base RT plans from *Institution E* covered only the whole breast, excluding the regional nodal areas in Step 1 (Fig. 3a and b). This institutional policy resulted in lower dose coverage of the regional nodal areas for *Institution E* in Step 1, compared to the other institutions (Fig. 2). In Step 2, however, *Institution E* followed the target area definition provided, prescribing the standard dose to both the whole breast and regional nodal areas, and most of their DVH parameters shifted into the IQR of the inter-institutional distribution. *Institution D* prescribed the LN boost dose to the entire regional nodal area up to the SCN in Step 1 (Fig. 3c), resulting in excessive irradiation to the regional nodal areas. In Step 2, *Institution D* followed the provided LN boost target volumes, adding a 5 mm PTV margin to the initial LN beds, and similarity among the 60 Gy_3.5_ 95% isodose lines in Scenario 2 significantly improved (Table 3; p-value = 0.031).

A multi-institutional study by the Korean Radiation Oncology Group (KROG 21–01) developed deep learning-based automatic segmentation (DLBAS) for OARs and CTVs in breast cancer, and verified its efficacy in producing consistent contours and improving inter-institutional contour agreement compared to commercially available atlas-based auto segmentation [18], [19]. However, their study primarily examined the impact of DLBAS on contouring and did not explore its impact on planning and delivery [19]. Although other studies have also demonstrated the potential of AI auto-contouring in multi-institutional studies, they mainly focused on contouring performance and interobserver variation [20], [21]. To the best of our knowledge, this is the first study that observed the effect of AI auto-contouring on inter-institutional variations in dose distributions.

Various efforts have been made to reduce inter-institutional dose variability in multi-institutional studies. The National Radiation Group (NRG)/RTOG 1005 multi-institutional breast cancer trial conducted a two-step QA process for institutional credentialing, consisting of: (1) planning for a benchmark case based on given contours and a documented protocol, and (2) a rapid review for the first planning submission. Even though the institutions underwent initial training in which benchmark case planning was repeated until credentialing was achieved, the subsequent rapid review demonstrated an initial QA pass rate of only 65% [22]. This may have been due to the institutions having to plan solely based on a written protocol for contouring and planning. In contrast, the AI auto-contouring engine in this dummy run provided tailored CTV/OARs.

This study has several limitations. The AI auto-contoured structures may be suboptimal for the diverse patient anatomies enrolled. Although the host institution allowed CTV modifications at participating institutions during this dummy run, a reduction in inter-institutional variations in dose/volume metrics was still observed. This suggests that even if the AI auto-contoured structures are not optimal for every patient, their use as reference structures would contribute to reducing inter-institutional variations in dose distributions. Furthermore, although axillary ultrasonography (US) and magnetic resonance imaging (MRI) are most accurate for LN treatment response evaluation after NAC, recent reviews have reported insufficient sensitivity (SE) and specificity (SP) for these modalities. The SE and SP of axillary US were estimated to be 65% and 69%, respectively. The corresponding values for MRI were 60% and 76%, respectively. Although F-fluorodeoxyglucose positron emission tomography-computed tomography (F-FDG PET-CT) has a relatively high SP of 86%, its SE of 38% remains a limitation [23], [24], [25], [26]. These limitations hinder the delineation of LN boost targets and may contribute to inter-institutional variations in dose distribution. Recent studies on radiomics, AI-based prediction models, and PET-MRI have demonstrated promising performance for imaging assessment of axillary response after NAC [27], [28], [29]. These emerging technologies will improve the precision of LN boost target delineation.

This dummy run study demonstrated that the provision of AI auto-contoured structures and LN boost target volume information reduced variation in dose distributions among institutions. Based on these findings, all participating institutions reached a consensus on the RT plan protocol, explicitly incorporating four key components: AI auto-contoured structure, LN boost target volume, target area definition, and OAR dose constraints. Establishing an RT plan protocol achievable for all institutions will contribute to the data reliability of RTaNAC. To support RTaNAC, the host institution has provided the customized version of the AI auto-contouring engine to all participating institutions to create tailored contours for each patient. Further, continuous communication through periodic joint meetings has ensured protocol compliance across the participating institutions. Consequently, this study has established a foundation for expanding RTaNAC into a multi-institutional investigation.

## Ethical approval

This study has been approved by the Institutional Review Board of the National Cancer Center and Cancer Research Institute in Korea (IRB no. NCC2023-0225, 08-13-2023). Informed consent was obtained from all subjects involved in the study.

## CRediT authorship contribution statement

**Wonhyeong Lee:** Writing – review & editing, Writing – original draft, Visualization, Software, Formal analysis, Data curation, Conceptualization. **Yeon-Joo Kim:** Writing – review & editing, Supervision, Resources, Project administration, Methodology, Investigation, Conceptualization. **Jin Hee Kim:** Writing – review & editing, Resources, Investigation. **Sung-Ja Ahn:** Writing – review & editing, Resources, Investigation. **Jong Hoon Lee:** Writing – review & editing, Resources, Investigation. **Younghee Park:** Writing – review & editing, Resources, Investigation. **Jin Hwa Choi:** Writing – review & editing, Resources, Investigation. **Jin-ho Song:** Writing – review & editing, Resources, Investigation. **Yoonsun Chung:** Writing – review & editing, Methodology.

## Funding

This work was supported by the National Cancer Center Korea [grant numbers 2311370, 2510772].

## Declaration of competing interest

The authors declare that they have no known competing financial interests or personal relationships that could have appeared to influence the work reported in this paper.

## Data Availability

Research data are stored in an institutional repository and will be shared upon request to the corresponding author.
