# Supplementary material for: Quality assurance incorporating artificial intelligence-generated reference contours in a phase II radiotherapy trial
Source: Phys Imaging Radiat Oncol. 2026 Mar 18;38:100949. doi: 10.1016/j.phro.2026.100949 (PMC13150357; doi:10.1016/j.phro.2026.100949)
Supplement: Supplementary Data 1 [file mmc1.pdf]

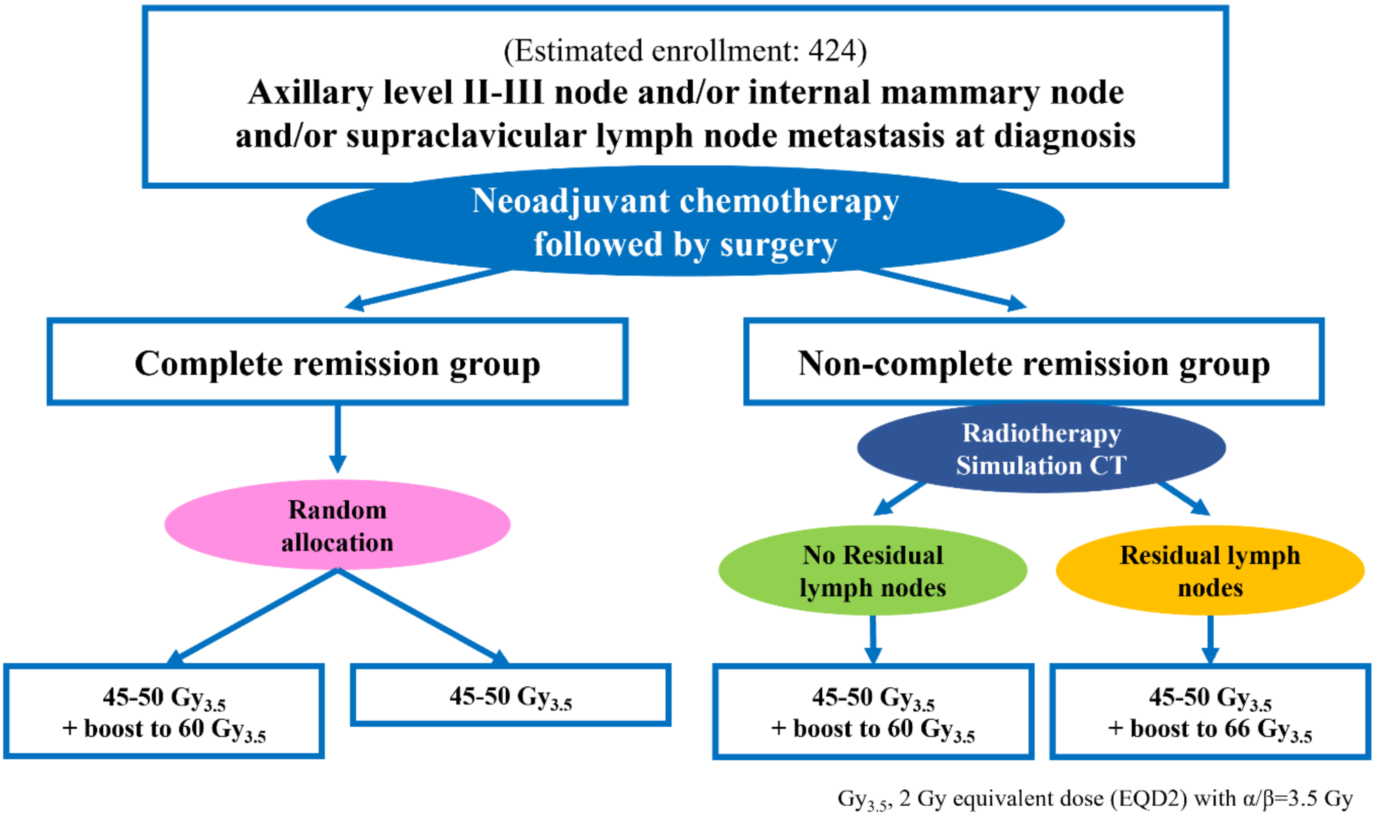

Supplementary Figure S1. Flow chart of the RTaNAC clinical trial.

Abbreviations: RTaNAC, Prospective phase II study of tailored radiotherapy according to the response after neoadjuvant chemotherapy followed by surgery in patients with lymph node metastasis in axillary level II-III and/or internal mammary and/or supraclavicular lymph node at diagnosis

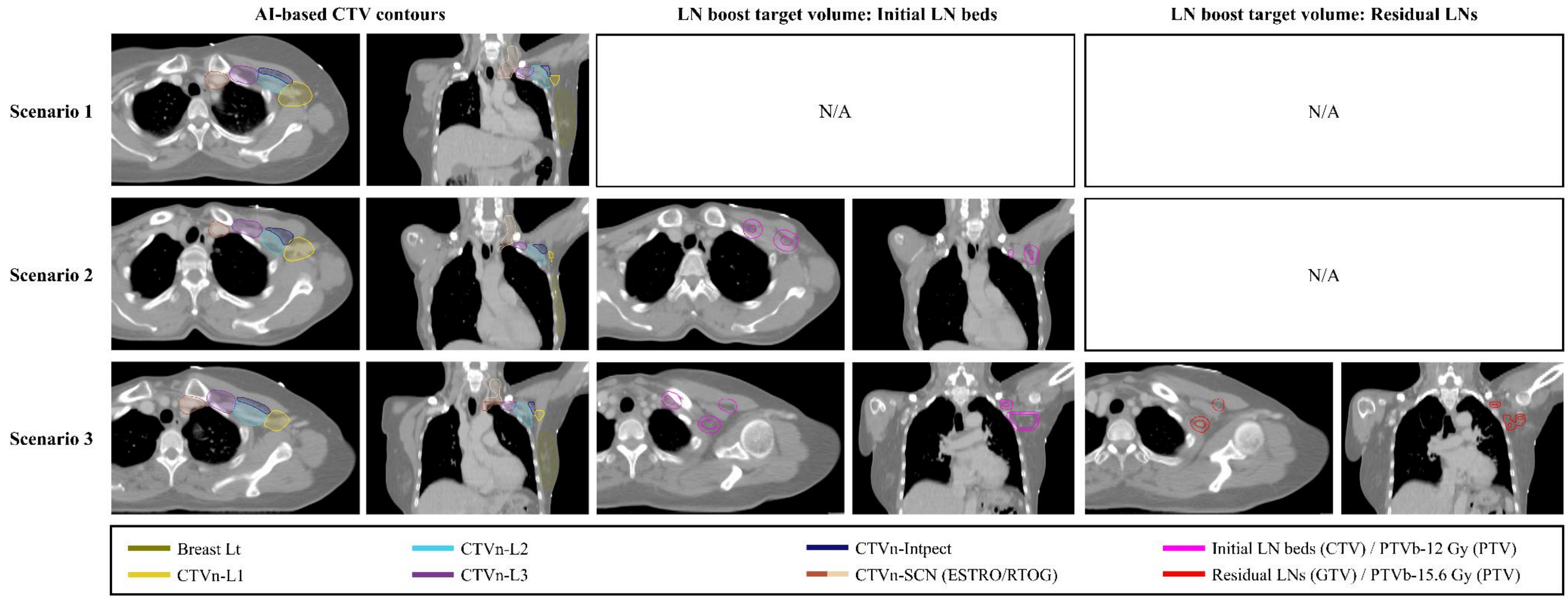

Supplementary Figure S2. Structures provided in Step 2. AI auto-contoured CTVs included Breast Lt, CTVn-L1-3, CTVn-Intpect, and CTVn-SCN (ESTRO/RTOG). LN boost target volumes were provided with 5-7 mm PTV margins from the initial LN beds and residual LNs, respectively.

Abbreviations: AI, artificial intelligence; CTV, clinical target volume; LN, lymph node; Lt, left; CTVn, nodal clinical target volume; L1-3, axillary level I-III; Intpect, interpectoral node; SCN, supraclavicular node; ESTRO, European Society for Radiotherapy and Oncology; RTOG, Radiation Therapy Oncology Group; PTVb, boost planning target volume; PTV, planning target volume; GTV, gross tumor volume

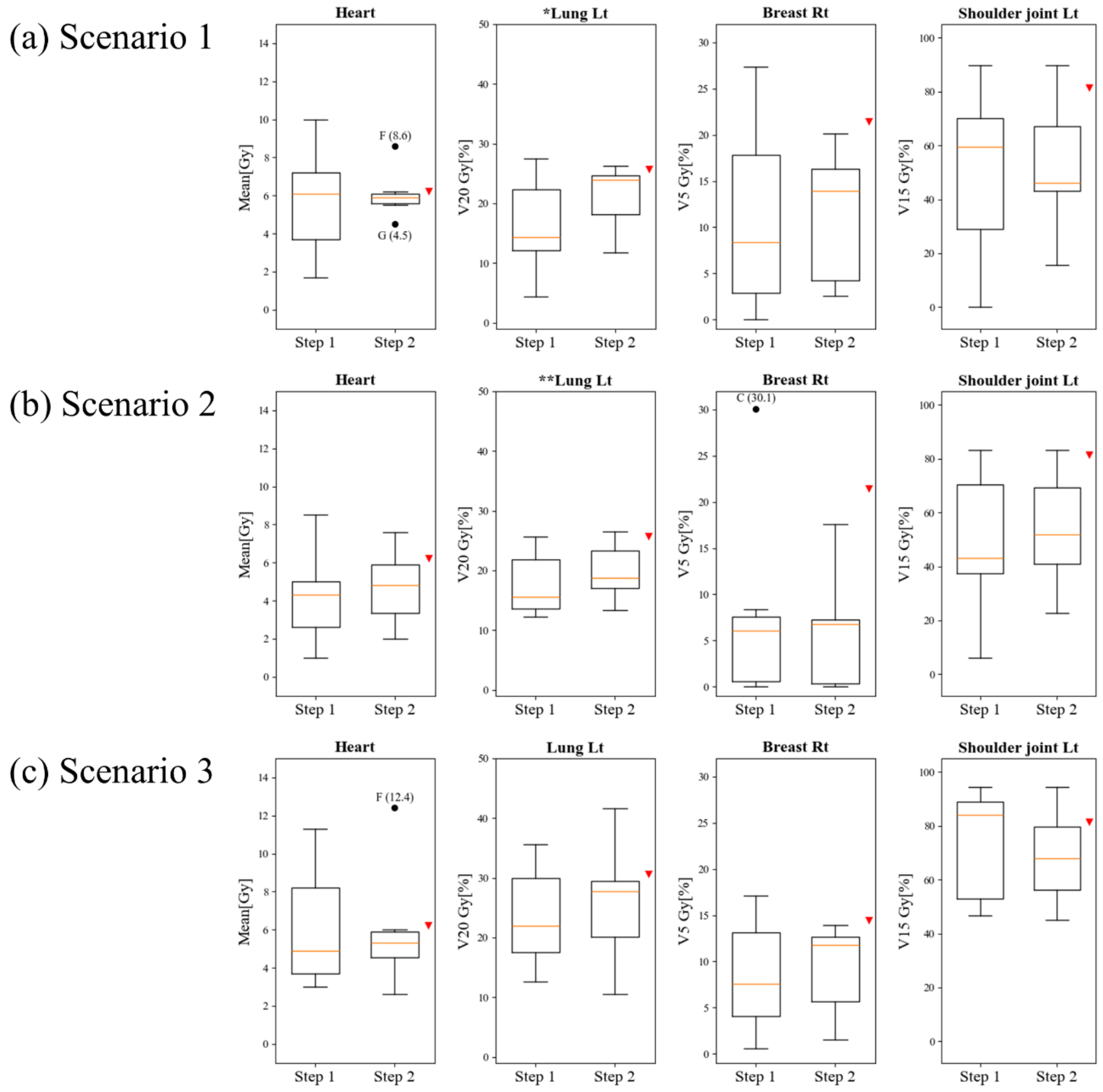

Supplementary Figure S3. Boxplots of dose-volume histogram parameter distributions in organs at risk (OARs) across the participating institutions for Step 1 and Step 2. (a) Scenario 1, (b) Scenario 2, and (c) Scenario 3.

The boxplots contain minimum/maximum, first (Q1)/third (Q3) quartile, and median values. Values that are more than 1.5 interquartile range (IQR, Q3 - Q1) away from Q1 or Q3 are defined as outliers, represented by a black dot (•) along with an institution identifier and corresponding value. Red inverted triangle symbol (▼) indicates the OAR constraint provided in Step 2. Single (*) and double (**) asterisks denote marginal (p-value < 0.1) and statistical significance (p-value < 0.05), respectively.

Abbreviations: V20 Gy, volume receiving 20 Gy; V5 Gy, volume receiving 5 Gy; V15 Gy, volume receiving 15 Gy; Lt, left; Rt, right
